# RCBVmax from DSC Perfusion MRI as a Supportive Imaging Biomarker for Differentiating IDH-Mutant Astrocytomas and Oligodendroglioma

**DOI:** 10.1007/s00062-026-01624-w

**Published:** 2026-02-18

**Authors:** Manoj Mannil, Christian Rubbert, Manfred Musigmann, Mathias Prokop, Paola Feraco, Frederick J. A. Meijer, Marion Smits, Anja G. Van der Kolk, Dylan Henssen

**Affiliations:** 1 Radboud university medical center, Nijmegen, Netherlands; 2 Münster University Medical Center, Münster, Germany; 3 Düsseldorf University Medical Center, Düsseldorf, Germany; 4 Münster University Medical Center, Münster, Germany; 5 Centre for medical sciences (CISMed), University of Trento, Trento, Italy; 6 University Medical Center Rotterdam, Rotterdam, Netherlands; 7 Erasmus MC Cancer Institute, Rotterdam, Netherlands; 8 Universitätsklinikum Leipzig, Leipzig, Germany

**Keywords:** Astrocytoma, Oligodendroglioma, Dynamic Susceptibility Contrast Perfusion MRI, rCBVmax

## Abstract

**Background:**

Gliomas, particularly astrocytomas (IDH-mutated, 1p/19q intact) and oligodendrogliomas (IDH-mutated, 1p/19q co-deleted), differ significantly in their clinical behavior and prognosis. Accurate differentiation between these subtypes is crucial for guiding therapeutic decisions. Non-invasive imaging biomarkers, such as T2-Flair mismatch sign and relative cerebral blood volume (rCBV), have shown promise in glioma classification.

**Methods:**

This retrospective multicenter study analyzed 42 patients with astrocytoma and 21 with oligodendroglioma. Demographic, histopathological, and imaging data were collected, with a focus on rCBV parameters derived from dynamic susceptibility contrast perfusion MRI. Statistical analyses were conducted to assess differences between the groups and evaluate the discriminatory power of rCBV metrics. After correction for multiple corrections, level of significance was set at *p* < 0.01.

**Results:**

Patients with astrocytoma and oligodendroglioma were similar in age and gender distribution (mean age and male-female-ratio; *p* = 0.01 respectively *p* = 0.04). The rCBVmax value was significantly higher in oligodendrogliomas (11.64 vs. 7.69, *p* < 0.001) and demonstrated a good discriminatory power (AUC = 0.764). Median and mean rCBV values showed non-significant differences between oligodendroglioma and astrocytoma (*p* = 0.02 respectively *p* = 0.03).

**Conclusion:**

rCBVmax emerges as a potential imaging marker for differentiating oligodendrogliomas from astrocytomas. These findings can be explained by biological properties of oligodendrogliomas and thereby underscore the potential of advanced imaging techniques in non-invasive glioma classification. However, larger prospective studies are required to validate these results and standardize imaging protocols.

## Introduction

Gliomas are the most common primary brain tumors, encompassing a spectrum of histological and molecular subtypes with distinct clinical behaviors and prognoses. Among these, astrocytomas (IDH-mutated, 1p/19q intact) and oligodendrogliomas (IDH-mutated, 1p/19q co-deleted) represent biologically and clinically diverse entities. Accurate differentiation between these subtypes is critical, as it directly informs treatment strategies, including the extent of surgical resection, radiotherapy, and chemotherapy regimens [1].

Differentiating these entities is particularly important due to their divergent prognoses and therapeutic responses. Astrocytomas, typically more aggressive than oligodendrogliomas, benefit from tailored surgical resection strategies aimed at maximal safe removal. Radiotherapy is often a cornerstone for astrocytomas, with the regimen varying based on tumor grade and patient condition. Oligodendrogliomas, in contrast, exhibit better responses to combined chemotherapy (e.g., procarbazine, lomustine, and vincristine [PCV]) and radiotherapy, attributed to their molecular profile. The 1p/19q co-deletion, often accompanied by IDH mutations, increases oligodendroglioma sensitivity to alkylating agents like PCV by removing DNA repair genes, slowing tumor growth, and altering metabolism, thereby reducing the tumor’s ability to withstand chemotherapy-induced damage. Failure to accurately distinguish these subtypes can lead to suboptimal treatment choices, affecting patient survival and quality of life [2].

The 2021 World Health Organization (WHO) classification of central nervous system tumors introduced significant advancements in the understanding and categorization of gliomas and takes into account the IDH mutation status and the presence of 1p/19q co-deletion. [3].

Traditional diagnostic approaches rely on histopathological examination and molecular profiling. However, these methods often necessitate invasive biopsy procedures, which carry inherent risks, especially in eloquent brain regions. Advanced imaging techniques, particularly dynamic susceptibility contrast (DSC) perfusion MRI, have emerged as valuable non-invasive tools for characterizing gliomas. DSC perfusion MRI measures hemodynamic parameters by monitoring changes in signal intensity during the passage of a bolus of contrast agent through the cerebral vasculature. Among these parameters, relative cerebral blood volume (rCBV) has shown particular utility. rCBV reflects the relative volume of blood within a given volume of brain tissue, serving as a marker of tumor vascularity and angiogenesis. Tumors with high rCBV are typically more aggressive and correlate with higher histological grades. DSC offers several advantages, including rapid acquisition, quantitative output, and the ability to assess heterogeneity within the tumor microenvironment. However, variations in imaging protocols and contrast administration can influence measurements, emphasizing the need for standardization across institutions [4].

This study explores the utility of rCBV parameters in distinguishing astrocytomas from oligodendrogliomas. By analyzing demographic, histopathological, and imaging data, we aim to identify a robust biomarker that enhances diagnostic accuracy and guide personalized treatment. Additionally, we propose avenues for future research, emphasizing the integration of imaging biomarkers into routine clinical practice to improve patient outcomes.

## Materials and Methods

A multicenter, retrospective study was conducted in The Netherlands and Germany. Patients diagnosed with either astrocytoma or oligodendroglioma between January 2022 and August 2024 were eligible for inclusion. Histopathological and molecular confirmation of the tumor type diagnosis was needed for inclusion. Furthermore, patients were included when DSC perfusion MRI data were available at the moment of lesion detection (i.e., patients did not yet undergo any neurosurgical intervention (including biopsy), radiotherapy and/or chemotherapeutic or anti-angiogenic therapies). Data on gender distribution, age, and histopathological subtypes were collected.

Ethical approval for this study was waived by our institutional review board because of the retrospective study design.

### DSC MRI and rCBV Analysis

Imaging was performed on 3T clinical MR imaging systems of two vendors (*Siemens Healthineers, Erlangen, Germany *and *Philips Healhcare, Eindhoven, The Netherlands*). DSC perfusion MRI was performed using a gradient-echo echoplanar imaging sequence. Prior to the dynamic study, a saturation pre-bolus of 25% of the total gadolinium-based contrast agent (GBCA) dose was administered in order to reduce contaminating T_1_ effects from contrast agent leakage. Dynamic T_2_*-weighted images were acquired during the first pass of a bolus of GBCA (0.1 mmol/kg body weight) at a rate of 2.5 ml/s to 3.0 ml/s. Imaging parameters were: repetition time (TR)/echo time (TE) 1,670/45 ms; field-of-view (FOV) 230 × 230 mm^2^; matrix 128 × 128; voxel size 1.8 × 1.8 × 5.0 mm^3^; interslice gap 30%; flip angle 90°; signal bandwidth 1346 Hz/x.

A dedicated workstation was equipped with OsiriX MD (Version 12.0; http://www.osirix-viewer.com) and a commercially available plug-in (IB Rad Tech; Imaging Biometrics, Elm Grove, Wisconsin) which uses automatic motion correction, image registration and a leakage-correction algorithm to process perfusion data and calculate perfusion maps [5–7]. This software package was used to assess the quantitative rCBV parameters, including median (rCBVmedian), mean (rCBVmean), and maximum (rCBVmax). These DSC perfusion metrics, as well as lesion volume, were derived from the entire lesions by use of a volume of interest (VOI) drawn semi-automatically and covering both the enhancing and non-enhancing elements of the lesions, though excluding large vessels [5, 8, 9]. Normalization of the cerebral blood volume map was automatically performed by placing reference VOIs following the methodology described by Bedekar et al. [10]. Both enhancing and non-enhancing tumor components were intentionally included within the VOI, as contrast enhancement in WHO grade 2 gliomas is often absent or heterogeneous and does not necessarily indicate malignant transformation, while their combined assessment enables rCBVmax to capture focal intratumoral angiogenic hotspots in a manner consistent with prior DSC-PWI literature and real-world preoperative imaging practice [11].

### Statistical Analysis

All statistical analyses were performed using R software (version 4.1.2). Normality of data distributions was assessed using the Shapiro-Wilk test. Features with normal distributions were further analyzed using Bartlett’s test for homogeneity of variances, followed by Student’s t‑tests. Non-normally distributed features were evaluated with the Wilcoxon (Mann-Whitney-U) test. Correction for multiple comparisons was carried out using the Bonferonni test, resulting in an adjusted level of significance at a *p*-value < 0.01.

To assess the discriminatory power of the rCBV features for distinguishing astrocytomas from oligodendrogliomas, receiver operating characteristic (ROC) curve analysis was conducted, and the area under the curve (AUC) values were calculated. The AUC is a measure of diagnostic accuracy, ranging from 0.0 to 1.0. An AUC below 0.6 indicates no or inadequate discriminatory ability. Values between 0.6 and 0.7 are considered acceptable, while an AUC of 0.7 to 0.8 is deemed good. AUC values between 0.8 and 0.9 indicate very good diagnostic performance, whereas a value higher than 0.9 reflects excellent accuracy [12, 13]. To determine the optimal cut-off points for the rCBV features, the Youden Index was calculated.

To account for potential confounding effects of demographic variables, a multivariable logistic regression analysis was performed including rCBVmax, age, and sex as covariates to assess the independent association of rCBVmax with tumor subtype. For this separate analysis, the level of significance was set at a *p*-value of < 0.05.

## Results

### Significantly Higher rCBVmax Values in Oligodendroglioma

**Fig. 1:**
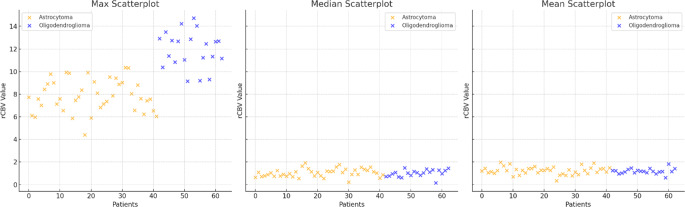
Scatterplots of rCBV Features Across Tumor Types

Our study cohort comprised 42 patients with a histopathologically confirmed grade 2 astrocytoma (IDH-mutated) and 21 patients with a confirmed grade 2 oligodendroglioma (IDH-mutated, 1p/19q co-deleted). The demographic and histopathological characteristics of the cohort are summarized in Table 1. Patients with astrocytoma were not significantly older than those with oligodendroglioma (mean age: 53.1 years vs. 44.0 years, *p* = 0.01). Within the astrocytoma group, the male-to-female-ratio was 2:1 and within the oligodendroglioma group, the male-to-female-ratio was 1.3:1 (*p* = 0.04). Quantitative analysis revealed that the astrocytoma group and the oligodendroglioma group had similar median rCBV (rCBVmedian; 1.08 vs. 0.94, *p* = 0.02) and mean rCBV (rCBVmean;1.31 vs. 1.23, *p* = 0.03) values. The maximum relative cerebral blood volume (rCBVmax), on the other hand, showed a significant difference between the groups (7.69 vs 0.11.64, *p* < 0.001) (Fig. 1).

**Table 1:** Demographic and Histopathological Characteristics

Characteristic	Astrocytoma (*n* = 42)	Oligodendroglioma (*n* = 21)	*p*-value
Male (%)	66.67	42.86	0.04
Female (%)	33.33	57.14	0.04
Mean Age (Years)	53.1	44.0	0.01
rCBV Median	1.08	0.94	0.02
rCBV Mean	1.31	1.23	0.03
rCBV Max	7.69	11.64	< 0.001

Although age and sex differences between the groups did not survive correction for multiple comparisons, numerical differences remained substantial. To further assess the impact of rCBVmax on distinguishing tumor subtypes, we performed a multivariable logistic regression analysis, adjusting for age and sex. The results indicated that rCBVmax remained a significant independent discriminator between grade 2 astrocytoma and oligodendroglioma. Specifically, for each unit increase in rCBVmax, the odds of having an oligodendroglioma as compared to an astrocytoma increased by a factor of 1.34 (Exp(B) = 1.34, *p* = 0.002). Age was also found to be a significant predictor (*p* = 0.034), with older age being associated with a higher likelihood of having an astrocytoma rather than an oligodendroglioma (Exp(B) = 0.944). In contrast, sex, rCBVmean or rCBVmedian did not reach statistical significance (*p* = 0.067, *p* = 0.667 and *p* = 0.470, respectively).

**Fig. 2:**
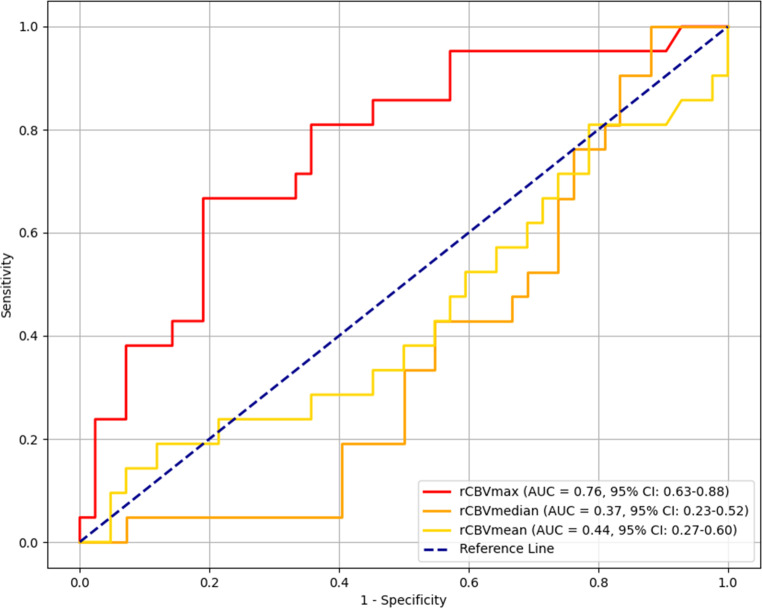
ROC curves and AUC analysis results of three rCBV measures to distinguish astrocytomas from oligodendrogliomas

ROC curve analysis demonstrated that rCBVmax had the highest univariate discriminatory power, with an AUC of 0.764, indicating good diagnostic accuracy to distinguish astrocytomas from oligodendrogliomas. Moderate discriminatory power was observed for rCBVmedian (AUC = 0.6315), while rCBVmean provided inadequate discriminatory capability (AUC = 0.5612). Figure 2 illustrates the corresponding ROC curves. The optimal cut-off points were 1.62 for rCBVmax (sensitivity: 78%, specificity: 83%), 1.13 for rCBVmedian (sensitivity: 72%, specificity: 55%), and 1.79 for rCBVmean (sensitivity: 68%, specificity: 66%).

## Discussion

Our study elucidates significant differences in rCBVmax value between astrocytoma and oligodendroglioma. More specifically, our study found that rCBVmax was significantly higher in oligodendrogliomas compared to astrocytomas with an AUC of 0.76. Importantly, rCBVmax remained independently associated with tumor subtype after adjustment for age and sex, supporting that the observed perfusion differences are primarily driven by underlying tumor biology, although age also contributed to distinguishing tumor subtypes. These findings align with existing literature suggesting that astrocytomas tend to present in older populations. The here reported findings can be explained by intrinsic tissue properties of low grade gliomas. Guo et al. noted that grade 2 oligodendrogliomas exhibit more dominant angiogenesis features as compared to grade 2 astrocytomas. More specifically, histopathological evaluation showed that oligodendroglioma has a higher microvascular density, larger vessel size and larger long axis diameter of vessels as compared to astrocytomas [14]. These features can help to explain the here reported differences in tumor perfusion as the rCBVmax reflects the relative peak microvascular blood volume. However, the results contradict previous results on the use of arterial spin labeling, another perfusion MRI technique, being unable to distinguish oligodendroglioma from astrocytoma [15]. Furthermore, Arévalo-Pérez et al. reported that dynamic contrast enhancement perfusion MRI was not capable of discerning oligodendroglioma from astrocytoma [16]. When focusing on DSC perfusion MRI, a some meta-analysis argue that the literature on the prediction of 1p/19q codeletion status is too limited [17]. However, Pons-Escoda et al. reported that oligodendrogliomas also exhibits significant higher perfusion metrics when using DSC perfusion MRI [18]. In contrast to prior DSC perfusion studies, the present work specifically focuses on rCBVmax rather than mean or median rCBV and exclusively analyzes molecularly defined WHO 2021 astrocytomas and oligodendrogliomas. Moreover, the multicenter, multi-vendor design employing leakage-corrected DSC workflows allows our findings to extend existing literature by demonstrating the robustness of rCBVmax-based subtype discrimination under real-world imaging conditions. Differences between DSC, ASL, and DCE perfusion techniques can be attributed to their distinct physiological sensitivities, with DSC primarily reflecting microvascular blood volume, whereas ASL and DCE probe different hemodynamic and permeability-related properties, which may explain discrepant findings across studies. Additionally, the T2-FLAIR mismatch sign has been proposed as a reliable MRI marker for distinguishing IDH-mutant astrocytomas from oligodendrogliomas, with high specificity but variable sensitivity. Although our study focused on perfusion metrics, combining rCBVmax assessment with T2-FLAIR mismatch evaluation could further improve non-invasive glioma subtype classification.

### Implications for Clinical Practice

Accurately identifying glioma subtype prior to biopsy or surgical resection is pivotal for guiding optimal treatment strategies and improving patient outcomes. Differentiating between astrocytomas and oligodendrogliomas has direct implications for surgical planning, as the extent and approach to resection differ significantly between these subtypes. For instance, astrocytoms may benefit from more extensive resection due to their typically less favorable prognosis, compared to oligodendrogliomas. The demonstrated statistically significant difference of rCBVmax as a non-invasive tool for preoperative assessment underscores its potential to refine diagnostic precision, thereby informing tailored surgical strategies. Moreover, reducing reliance on invasive biopsy procedures is particularly advantageous in cases where surgical risks are high. Routine integration of rCBV metrics with molecular profiling (e.g., IDH mutation status and 1p/19q co-deletion) could further enhances the diagnostic framework, enabling a more nuanced and patient-specific approach to glioma management. rCBVmax should be regarded as a complementary, group-level imaging biomarker that supports glioma subtype assessment when interpreted alongside conventional MRI features and molecular information, particularly in the preoperative setting where tumor grade is often unknown.

### Limitations

Despite its strengths, this study has several limitations. First, the retrospective nature of the study introduces selection bias, as only patients with complete imaging and histopathological data were included; our findings are therefor at present not valid for patients with a suspected glioma prior to surgery. Second, the sample size, particularly for oligodendrogliomas, was relatively small, which may limit the generalizability of our findings. Future studies with larger, unselected cohorts and prospective designs are warranted to validate the observed trends. Third, while the rCBVmax parameter showed a discriminatory effect on the group level, its accuracy may vary depending on imaging protocols and post-processing techniques. Standardization of imaging methodologies across institutions would be essential for broader clinical implementation. VOIs were generated using a standardized semi-automated workflow with predefined intensity thresholds and manual correction by experienced neuroradiologists; although formal interobserver variability was not assessed, this approach was chosen to minimize subjective bias and is acknowledged as a limitation. The analysis was deliberately restricted to WHO grade 2 tumors to isolate molecular subtype-related perfusion differences independent of histological grade, as inclusion of grade 3 gliomas—despite occasional faint or patchy enhancement in a minority of cases—would have introduced a strong confounding effect given their known association with increased perfusion, with all diagnoses confirmed histopathologically and molecularly according to WHO 2021 criteria. One of the strengths of the present study is the multicenter, multinational approach. However, vendor- and protocol-related variability may influence absolute rCBVmax values and limit cross-site reproducibility, underscoring the need for standardized acquisition and post-processing protocols to facilitate broader clinical implementation.

### Future Directions

Building on these findings, future research should focus on integrating advanced imaging biomarkers with molecular diagnostics to develop comprehensive classification algorithms for gliomas. Prospective multicenter studies with standardized imaging protocols could further substantiate the role of rCBVmax in glioma differentiation. Additionally, exploring the prognostic implications of rCBV metrics and their association with treatment outcomes could provide valuable insights into their utility beyond diagnosis. The incorporation of artificial intelligence and machine learning techniques to analyze multimodal data could also enhance the diagnostic accuracy and predictive power of these biomarkers.

In conclusion, this study highlights the potential of rCBVmax as a non-invasive imaging marker for differentiating astrocytomas and oligodendrogliomas. While further research is needed to validate these findings and address existing limitations, rCBVmax should be regarded as a supportive, group-level imaging biomarker that complements conventional MRI features and molecular diagnostics rather than providing individual-level diagnostic certainty.

## Data Availability

No datasets were generated or analysed during the current study.
